# Development of an Integrated Screening Framework for Marine-Derived *Bacillus* Probiotics

**DOI:** 10.3390/md24040137

**Published:** 2026-04-15

**Authors:** Yaoying Lu, Xiaojing Chen, Yunjiang Feng

**Affiliations:** 1 Institute for Biomedicine and Glycomics, Griffith University, Parklands Drive, Gold Coast, QLD 4222, Australia; y.lu@griffith.edu.au; 2 Bioproton Pty Ltd., 55 Dulacca St., Brisbane, QLD 4110, Australia; wendy@bioproton.com; 3 School of Environment and Science, Griffith University, 170 Kessels Road, Brisbane, QLD 4111, Australia

**Keywords:** marine-derived *Bacillus*, probiotics, antimicrobial, screening workflow

## Abstract

Probiotics are known to improve gut microbiota balance, enhance food digestion, and support overall health. Among these, *Bacillus* species are particularly promising due to their safety, spore-forming ability, environmental resilience, and diverse enzymatic activities. However, most *Bacillus* probiotics used in industry are of terrestrial origin, leaving marine-derived strains largely unexplored. Utilising the untapped potential of marine microbial biomass, this study presents a multi-stage methodology for identifying and evaluating marine-derived *Bacillus* strains with probiotic potential. A structured screening pipeline was applied to 67 microbial isolates from the Great Barrier Reef sponges. Initial selection focused on essential probiotic characteristics, including growth, stability, safety, and survival under gastrointestinal conditions. Strains meeting these criteria were then assessed for desirable properties, including digestive enzyme production and pathogen inhibition. Using this workflow, three marine-derived *Bacillus* strains were identified as potential probiotics, one of which demonstrated strong antimicrobial activity against *Salmonella enterica* at 5 and 10 mg/mL (*p* < 0.01). These findings demonstrate the capability of marine-associated *Bacillus* as novel bioproducts with functional antimicrobial properties.

## 1. Introduction

Probiotics are defined as “live microorganisms which when administered in adequate amounts confer a health benefit on the host” [1]. They are known to improve gut microbiota balance, support food digestion, and suppress pathogenic microorganisms [2,3]. Among various probiotic groups, *Bacillus* species (*Bacillus* spp.) are particularly promising due to their strong safety record [4,5] and their ability to form highly resilient endospores, enabling survival under harsh gastrointestinal (GI) and environmental conditions [6]. Additionally, *Bacillus* spp. produce a wide range of digestive enzymes and antimicrobial compounds, supporting nutrient utilisation and helping to suppress harmful pathogens [7,8].

*Bacillus* probiotics used in animals are mainly derived from *B. subtilis* strains isolated from host-associated sources, environmental habitats (soil, ponds, or seaweed), and fermented foods [9]. In contrast, marine environments harbour microbial communities that are compositionally distinct from those on land, even though their overall diversity is comparable [10]. Among marine habitats, sponges (phylum Porifera) are important sources of marine microorganisms. Their high seawater filtration rates and nutrient-rich tissues create stable microenvironments that support diverse and abundant microbial communities, with microorganisms comprising up to one-third of the sponge’s biomass [11,12,13]. These sponge-derived microorganisms produce novel enzymes and bioactive compounds, making them especially valuable for biotechnology and probiotic discovery [14,15,16].

Despite this biotechnological potential, the identification of sponge-derived probiotics for animal nutrition requires that candidate microorganisms meet both safety and regulatory standards. The Association of American Feed Control Officials (AAFCO) and the European Food Safety Authority (EFSA) provide strict criteria and approved strain lists for microbial feed additives, ensuring that probiotic strains used in animal feed are safe and compliant [4,5].

The NatureBank (www.griffith.edu.au/research/institute-biomedicine-glycomics/facilities/naturebank accessed on 19 March 2026) at the Institute for Biomedicine and Glycomics, Griffith University, comprises a microbial collection of over 2000 marine-derived microbial isolates. These microorganisms were predominantly isolated from sponges collected at the Great Barrier Reef [17]. This unique resource provides an opportunity to explore the probiotic potential of marine-derived *Bacillus* strains. The aim of this work was to develop an integrated screening framework to identify and evaluate marine-derived *Bacillus* strains with probiotic potential. The workflow first assessed essential probiotic characteristics, including growth performance, stability, safety, and gastrointestinal tolerance. It was followed by the evaluation of functional properties such as digestive enzyme production and antimicrobial activity. Using this pipeline to screen 67 sponge-derived isolates, we identified three safe *Bacillus* strains suitable for further development, including one with significant antimicrobial activity.

## 2. Results

### 2.1. Source of Marine-Derived Microorganisms and Screening Strategies

**Figure 1:**
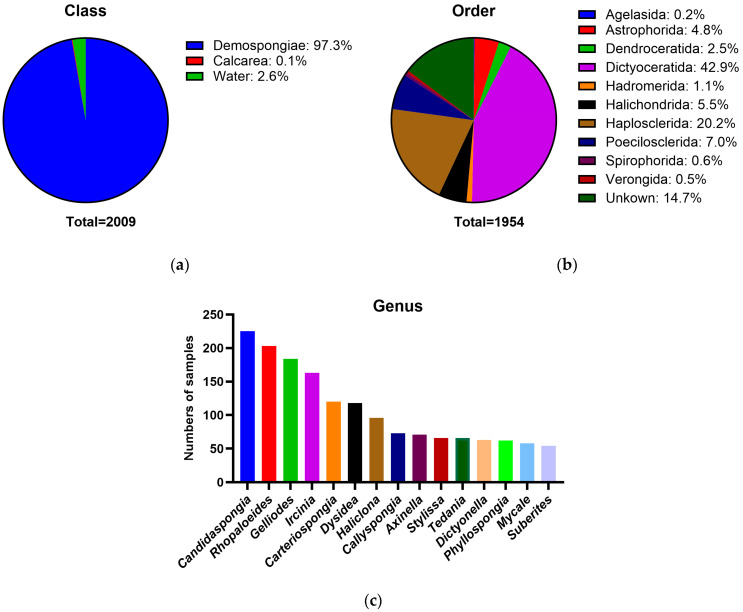
Host taxonomic composition of sponge specimens associated with the microbial collection. (**a**) Class-level distribution of sample sources, showing sponge hosts belonging to the classes Demospongiae and Calcarea, alongside seawater samples; (**b**) Order-level taxonomic distribution of Demospongiae host sponges; (**c**) The 15 most abundant sponge host genera from which microorganisms were isolated.

An extensive collection of over 2000 unknown marine-derived microorganisms was used in this study. These microbial isolates mainly originated from sponges collected from the Great Barrier Reef [17]. Host taxonomic information was recorded for the source sponges from which microbial isolates were derived. The collection comprised 2009 microorganisms, and their origins are dominated by the class Demospongiae (*n* = 1954), with a minor contribution from Calcarea (*n* = 3), alongside 52 seawater samples (Figure 1a). At the order level, Demospongiae host sponges were primarily represented by Dictyoceratida, Haplosclerida, Poecilosclerida, Halichondrida, and Astrophorida (Figure 1b). In total, 27 host families and 163 host genera were identified within Demospongiae. The 15 most abundant sponge host genera contributing to the microbial collection are shown in Figure 1c, with *Candidaspongia*, *Rhopaloeides*, *Gelliodes*, *Ircinia,* and *Carteriospongia* representing the most abundant host sponge genera. Species-level host identification was available for 875 sponge specimens, while the remaining lacked confident species assignments. The sponge samples were processed, and bacterial isolates were cultured on marine-based and selective agar media and then cryopreserved at −80 °C [18]. Colony morphology information and cryopreservation conditions were recorded for each isolate. No taxonomic information was available for these 2009 sponge-derived microbial isolates.

### 2.2. Screening Strategies

**Figure 2:**
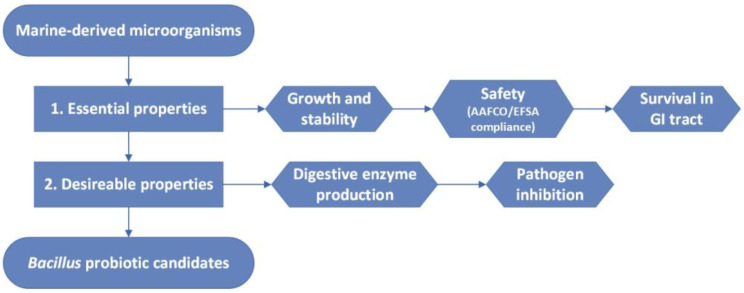
Sequential screening workflow for marine-derived *Bacillus* probiotic candidates as animal feed additives. Marine-derived microorganisms were first screened for essential probiotic properties, including growth performance, stability, safety (AAFCO/EFSA compliance, including haemolysis, antimicrobial susceptibility, and taxonomic safety), and gastrointestinal survival. Next, isolates meeting these requirements were retained and evaluated for desirable properties, such as extracellular digestive enzyme production and pathogen inhibition. Lastly, isolates meeting all requirements were retained as *Bacillus* probiotic candidates.

This study aimed to identify marine-derived *Bacillus* spp. that align with AAFCO or EFSA-approved strain lists for use as animal feed additives. Accordingly, microorganisms with circular or irregular colony morphology, characteristic of *Bacillus*, were randomly selected from the collection to screen for potential probiotics. A multi-step screening workflow was developed (Figure 2). Initial selection focused on essential probiotic characteristics, including growth performance, stability, safety, and gastrointestinal survival. Strains meeting these criteria were assessed for desirable properties, including digestive enzyme production and pathogen inhibition.

The workflow also integrated AAFCO and EFSA guidelines, ensuring that the marine-derived isolates not only exhibit essential and desirable probiotic properties but also align with established regulatory requirements for animal feed additives, thereby enhancing their commercial relevance.

### 2.3. Screening for Essential Probiotic Properties

#### 2.3.1. Growth and Stability

**Figure 3:**
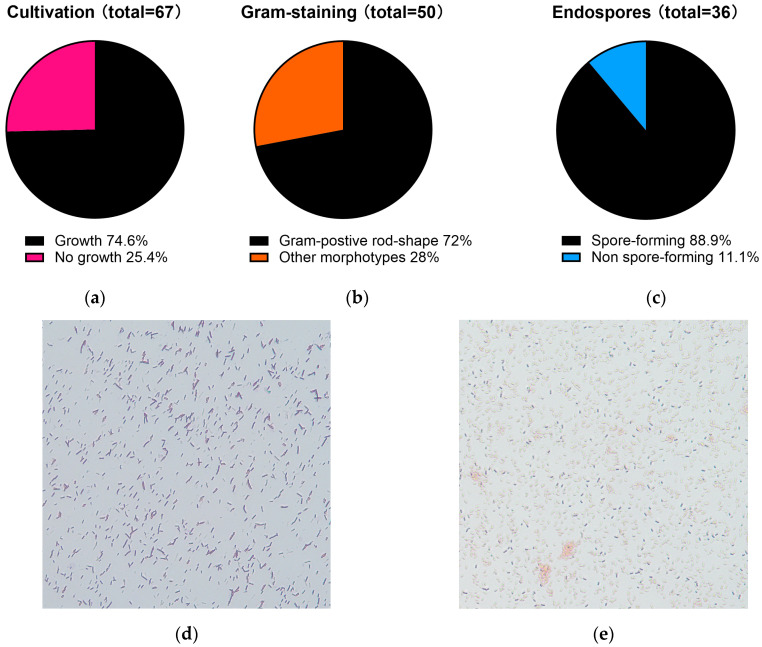
Distribution of marine-derived isolates meeting the growth and stability criteria. Marine-derived microbial isolates were sequentially screened under *Bacillus*-favourable cultivation conditions (37 °C for 16 h). (**a**) Under these conditions, 74.6% of isolates (50) exhibited rapid growth; (**b**) Growth-positive isolates were subjected to Gram staining and morphological analysis, showing that 72% (36) were Gram-positive rod-shaped bacteria; (**c**) Gram-positive rods were further evaluated for endospore-forming ability, with 32 isolates (88.9%) confirmed to form endospores; (**d**) Representative image of Gram-positive rod-shape marine-derived isolates under microscopy; (**e**) Representative image of a spore-forming marine-derived isolate under microscopy, with endospores in green colour. Images were cropped to enhance visualisation of spore morphology while preserving original staining characteristics.

Sponges from the Great Barrier Reef typically inhabit waters with temperatures ranging from 22 to 28 °C, while experimental evidence shows that disruption of the sponge–microbial symbiosis occurs when seawater temperatures approach 33 °C [19]. Therefore, microorganisms derived from these sponges are expected to exhibit optimal growth within this temperature range in seawater. In contrast, typical *Bacillus* spp. generally exhibit optimal growth at higher temperatures (25–37 °C) [20]. Therefore, *Bacillus* favourable cultivation conditions were applied for the initial screening phase. A total of 67 sponge-derived bacterial isolates were cultivated under *Bacillus* favourable conditions (37 °C for 16 h), resulting in 50 isolates (74.6%, Figure 3a) that exhibit rapid growth. These isolates were subsequently subjected to Gram staining, and 36 out of 50 isolates (72%, Figure 3b) were identified as Gram-positive and rod-shaped bacteria (Figure 3d), which is consistent with typical *Bacillus* morphology. Endospore production was then confirmed using both Schaeffer–Fulton spore staining and heat-treatment (60 °C for 30 min). A total of 32 isolates (out of 36 Gram-positive rods, Figure 3c) demonstrated clear endospore formation (Figure 3e). They also remained stable at 60 °C, a temperature at which spores are mostly active without reducing germination efficiency [21]. Therefore, these isolates were retained for further characterisation.

#### 2.3.2. Safety Tests

**Figure 4:**
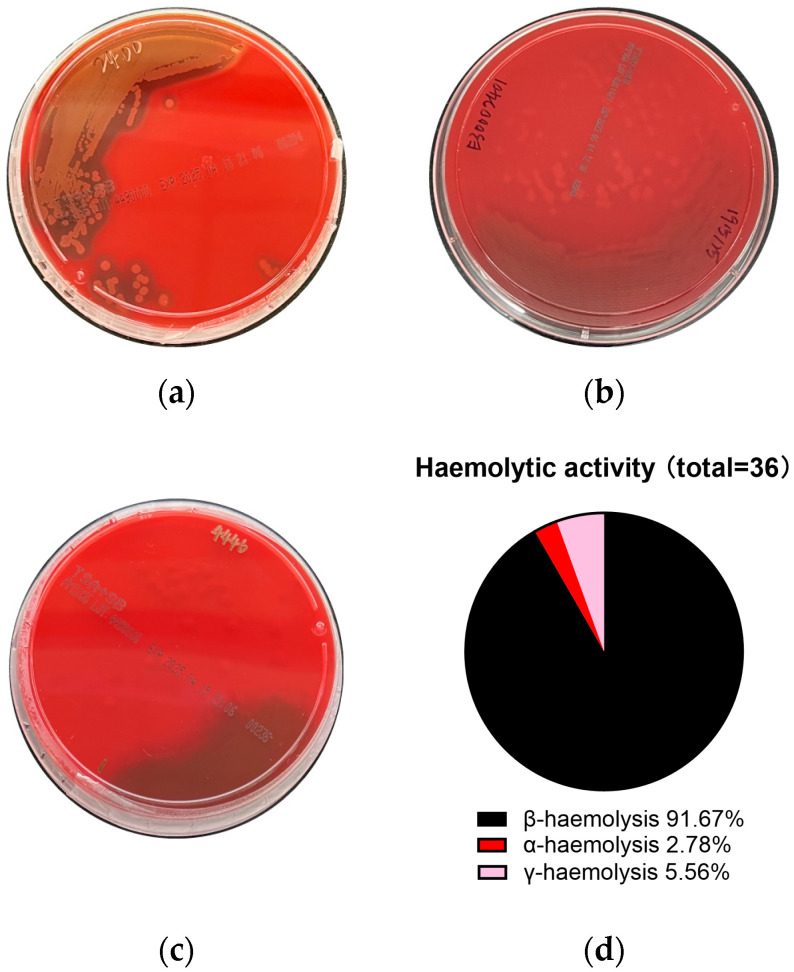
Haemolytic activity of candidate isolates on sheep blood agar. Marine-derived isolates were inoculated onto sheep blood agar and incubated at 37 °C for 16–24 h. Examples of haemolytic activity were shown: (**a**) β-haemolysis; (**b**) α-haemolysis; (**c**) γ-haemolysis; (**d**) Distribution of marine-derived isolates showing different haemolytic activity.

The marine-derived microorganisms were next evaluated for safety, beginning with a haemolysis test to determine their potential to lyse red blood cells. Haemolytic activity is commonly associated with toxicity and pathogenicity, such as streptolysin O and S produced by Group A Streptococcus (GAS), which contribute to β-haemolysis [22]. In contrast, non-haemolytic (γ-haemolysis) strains are preferred for human or animal use [23,24]. Haemolytic activity was assessed using sheep blood agar [25]. The presence of a clear zone surrounding bacterial colonies indicated β-haemolysis (Figure 4a), while partial haemolysis (greenish discolouration) and the absence of haemolysis corresponded to α- and γ-haemolysis (Figure 4b,c), respectively. Among the 36 isolates tested, 91.67% exhibited β-haemolysis, one isolate displayed α-haemolysis (2.78%), and two isolates showed γ-haemolysis (5.56%) (Figure 4d). Based on these results, only three isolates, which were non-haemolytic or exhibited weak haemolytic activity, were considered suitable for further assessments. They are abbreviated as candidates 1–3.

The second safety assessment is antimicrobial susceptibility testing. According to EFSA, microbial feed additives should not add to the pool of antimicrobial resistance (AMR) genes already present in the gut bacterial population. To assess this, two types of data are required. Firstly, phenotypic testing is based on the determination of a minimum inhibitory concentration (MIC) for a selected group of antimicrobials. Secondly, genomic analysis for the presence of known AMR genes [26].

In this study, phenotypic MIC testing was performed for candidates 1–3 against a panel of antimicrobials, including vancomycin, gentamycin, kanamycin, streptomycin, erythromycin, tetracycline, and chloramphenicol. The MIC was determined by OD_600_ measurements and compared with the EFSA-recommended cut-off thresholds. As summarised in Table 1, the MIC values of candidates 1–3 for all tested antimicrobials were equal to or below the EFSA-recommended cut-off thresholds, indicating susceptibility and the absence of phenotypic resistance. Based on these results, candidates 1–3 were phenotypically safe, but confirmation of their genomic AMR status will require future whole-genome analysis.

#### 2.3.3. Survival in Gastrointestinal (GI) Tract

Tolerance to acidic pH and bile salts is a key characteristic for probiotics to survive passage through the GI tract and exert functional effects in the host [27,28,29,30]. To evaluate GI stress tolerance, candidates 1–3 and five industrial benchmark *Bacillus* probiotic strains were evaluated under simulated gastrointestinal conditions.

Gastric pH varies widely among humans and animals, typically ranging from 1 to 3 in scavengers and carnivores, from 2.5 to 6.5 in herbivores, and around 2 to 4.5 in omnivores, depending on species and diet [31]. For this study, exposure to pH 4 was selected to mimic the acidic conditions encountered during gastric transit, providing a mild physiologically relevant stress capable of discriminating acid-tolerant strains. Bile salts are compounds synthesised from cholesterol in the liver, stored in the gallbladder, and released into the small intestine, where they are key determinants of the intestinal physicochemical environment [32]. To be effective, probiotics must survive this bile salt-rich environment. Therefore, this study used a bile salt concentration of 0.3% (*w*/*v*), which is widely adopted as a critical threshold for probiotic screening [33], to select bile salt-resistant probiotic strains.

Survival under acidic and bile conditions was assessed at 0, 2, and 4 h, corresponding to progressive exposure times during GI transit. For benchmark strains, survival at pH 4 ranged from 70 to 100% and 67 to 100% after 2 and 4 h of exposure, respectively (Table 2). All three candidates exhibited survival within this range. Under 0.3% bile salt stress, benchmark strains showed survival rates of 15–99% at 2 h and 12–98% at 4 h (Table 3). All three candidates displayed comparable bile tolerance to benchmark strains.

To confirm the taxonomic identity of the three probiotic candidates, the 16S rRNA gene of candidates 1–3 was sequenced by Sanger sequencing. Comparison against reference databases showed that all three isolates clustered within the genus *Bacillus* (Supplementary Tables S1–S3). These verified marine-derived *Bacillus* isolates were then evaluated for additional desirable probiotic properties.

### 2.4. Screening for Desirable Probiotic Properties

#### 2.4.1. Digestive Enzyme Production

*Bacillus* spp. are known to secrete extracellular digestive enzymes, such as proteases and amylases, which contribute to their ability to hydrolyse proteins and carbohydrates [8]. These enzymatic activities not only support nutrient acquisition but also strengthen their use as probiotics and industrial biocatalysts in food, feed, and biotechnological applications. Accordingly, screening for hydrolytic enzyme production was employed as a functional selection criterion to further prioritise isolates with potential applied functionality. All three candidates produced protease, while candidates 1 and 3 were additionally capable of amylase production (Supplementary Figure S1).

#### 2.4.2. Antimicrobial Activity

Probiotics with antimicrobial activity not only enhance host health but also help reduce reliance on antibiotics. It is a critical functional trait, reflecting the ability of probiotic candidates to inhibit pathogenic microorganisms in the gastrointestinal environment. The antimicrobial potential of selected *Bacillus* candidates was evaluated against five pathogens, including *Escherichia coli* (*E. coli*), *Salmonella enterica* (*S. enterica*), *Staphylococcus aureus* (*S. aureus*), and *Clostridium perfringens* (*C. perfringens*), which are major zoonotic and foodborne pathogens [34], and *Pseudomonas aeruginosa* (*P. aeruginosa*), an opportunistic pathogen that can cause severe infections in various livestock and poultry species [35,36]. Together, these pathogens represent both Gram-negative and Gram-positive bacteria commonly encountered in gastrointestinal and environmental settings.

Agar diffusion assay showed that only candidates 2 and 3 exhibited antimicrobial activity, with candidate 2 inhibiting *S. enterica* and candidate 3 inhibiting both *S. enterica* and *E. coli* (Supplementary Figure S2). Because this method provides only qualitative evidence, quantitative assessment was performed using broth microdilution assays.

**Figure 5:**
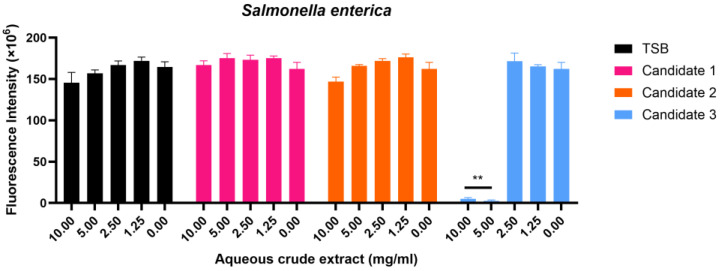
Broth microdilution assay of aqueous crude extracts from *Bacillus* candidates 1–3 against pathogenic bacteria. *S. enterica* were cultured in MHB and incubated with aqueous crude extracts of TSB, and candidates 1–3 at 37 °C for 16 h. The growth of pathogenic bacteria was then assessed using Resazurin, and fluorescence intensity was measured. Mean ±SEM (*n* = 3) of three or four independent experiments, each performed in triplicate, is shown. One-way ANOVA followed by Dunnett’s test was employed to compare treated cells with untreated cells for each extract group. *p* ** < 0.01.

To evaluate both water-soluble and organic-soluble secondary metabolites, antimicrobial activity was tested using aqueous crude extracts and ethyl acetate (EtOAc) extracts from candidates 1–3. Among three crude aqueous extracts, only candidate 3 exhibited significant inhibition against *S. enterica* at 5 and 10 mg/mL (Figure 5, *p* < 0.01) and showed a MIC of 5 mg/mL, whereas TSB control, candidate 1, and candidate 2 showed no effects (Figure 5). None of the aqueous crude extracts from any candidate exhibited activity against the remaining four pathogens tested (Supplementary Figure S3). Similarly, none of the EtOAc crude extracts showed activity against any of the five pathogens (Supplementary Figure S4).

Overall, candidate 3 consistently demonstrated selective antimicrobial activity against *S. enterica* across all assays, confirming that this strain produces bioactive compounds capable of suppressing *S. enterica* growth.

## 3. Discussion

This study established an integrated workflow for identifying marine-derived *Bacillus* strains with probiotic potential. From 67 sponge-derived isolates, three safe *Bacillus* strains were identified, including one with significant, selective *S. enterica* inhibition. This represented a 4.5% hit rate for utilising marine microorganisms for probiotic discovery. The workflow also provided a cost-effective pipeline for the discovery of novel marine-derived probiotics for animal feed additives. Together, these findings expand the underexplored potential of marine microbial resources and demonstrate the capability of marine-associated *Bacillus* as novel bioproducts with functional antimicrobial properties.

*Bacillus* probiotics used in animals are mainly derived from *B. subtilis* strains originating from host-associated sources (intestine and faeces), environmental habitats (soil, ponds or seaweed), and fermented foods [9]. Despite this broad range of terrestrial and host-associated origins, marine-derived *Bacillus* strains remain largely unexplored. The results of this study, therefore, expand current knowledge by demonstrating that sponge-associated marine microorganisms also harbour *Bacillus* strains with probiotic potential. Marine sponges are known to contribute to diverse bioactive compounds [37], and microorganisms from marine sponges may possess unique enzymatic profiles, bioactive metabolites not commonly found in terrestrial and host-associated *Bacillus*, thereby contributing to distinct functional probiotic properties. Moreover, sponge-derived microorganisms are naturally adapted to a wide range of marine environments characterised by fluctuating salinity, temperature, nutrient availability, and microbial competition [38]. These adaptive traits are highly relevant to aquaculture systems, where probiotic candidates need to remain functional and stable under dynamic rearing conditions. Consequently, sponge-associated bacteria represent a promising and underexplored source of probiotic strains with potential applications in aquaculture, including improving animal health, enhancing feed utilisation, and supporting disease management strategies.

Among the three *Bacillus* probiotic candidates, only the aqueous crude extract of candidate 3, derived from the cell-free supernatant, exhibited antimicrobial activity. This extract selectively inhibited *S. enterica*, which is the only Gram-negative bacterium among the five tested pathogens (Figure 5, *p* < 0.01). This anti-salmonella activity is likely derived from the water-soluble metabolites produced by candidate 3. *Bacillus* spp. are known to produce bioactive metabolites that can contribute to antimicrobial activity [39,40]. Further isolation and structural characterisation of the bioactive metabolites will require chromatographic (HPLC) and spectroscopic techniques (LC–MS/MS and NMR).

For further commercial development and to elucidate the mode of action, additional investigations such as colonisation assays, large-scale fermentation, feed formulation, animal trials, and comprehensive genomic and metabolomic analyses will be required. Additionally, whole-genome analysis is needed to assess the presence of AMR genes, to provide species or strain-level taxonomic resolution, and to identify genomic features associated with functional properties.

## 4. Materials and Methods

### 4.1. Chemicals and Reagents

All chemicals and reagents used in this study were sourced from commercial suppliers. Tryptic soy broth (TSB), resazurin, Gram staining kits, Schaeffer and Fulton spore stain kits, bile salts, vancomycin hydrochloride, streptomycin sulphate, erythromycin, tetracycline, gentamicin solution, and kanamycin solution were obtained from Sigma-Aldrich/Merck (Melbourne, Australia). Mueller–Hinton broth (MHB), nutrient broth, bacteriological agar (Agar No. 1), tryptone, yeast extract, and dextrose were purchased from Oxoid (Thebarton, Australia). Tryptic Soy Agar (TSA) plates supplemented with 5% sheep blood were supplied by Thermo Fisher Scientific (Thebarton, Australia). Ethyl acetate, methanol, KCl, MgSO_4_·7H_2_O, KH_2_PO_4_, Ca(NO_3_)_2_, MnCl_2_, FeSO_4,_ and chloramphenicol were obtained from ChemSupply (Gillman, Australia).

### 4.2. Microbial Strains

Sixty-seven marine-derived microorganisms were obtained from the NatureBank at the Institute for Biomedicine and Glycomics, Griffith University (Australia). Industrial benchmark strains: BPR-11 (*Bacillus amyloliquefaciens*, CBS#141692), BPR-14 (*B. amyloliquefaciens*, CBS#142360), BPR-16 (*B. velezensis*, CBS#148295), BPR-17 (*B. amyloliquefaciens*, CBS#148296), and BPR-20 (*B. subtilis*, CBS#144669) were obtained from Bioproton Pty Ltd. (Brisbane, Australia) [41]. The Centraalbureau voor Schimmelcultures (CBS) number was generated by Westerdijk Fungal Biodiversity Institute, Utrecht, The Netherlands. Pathogenic strains: *C. perfringens* (ATCC 13124), *E. coli* (ATCC 43887), *P. aeruginosa* (ATCC 10145), *S. aureus* (ATCC 6538), and *S. enterica* (ATCC 6960) were purchased from In Vitro Technologies (Melbourne, Australia).

### 4.3. Microbial Cultivation

Microorganisms were first streaked onto tryptic soy agar (TSA) plates and were incubated at 37 °C for 16–20 h in a static incubator. A single colony was then selected and inoculated into TSB, followed by incubation at 37 °C for 16–20 h at 200 rpm. Cultivation conditions followed standard microbiological procedures unless otherwise specified.

### 4.4. Gram Staining

Smears were prepared from a single bacterial colony or liquid culture on the microscopy slide. Gram staining was performed following the manufacturer’s instructions using crystal violet, iodine, decolourizer, and safranin. Slides were air-dried and examined under an Olympus BX63 microscope (Evident Scientific, Sydney, Australia) to assess cell morphology and Gram reaction.

### 4.5. Sporulation Stimulation and Verification

A single colony was inoculated into TSB and incubated overnight at 37 °C, 200 rpm until reaching the stationary growth phase. Then, 1–3% of the overnight culture was transferred into sporulation medium (Table S4), and the cultures were incubated at 37 °C for 48–72 h. Spore formation was then confirmed using the Schaeffer and Fulton spore stain kit (Merck, Melbourne, Australia), with spores appearing green and vegetative cells red or pink. The stability of spores was further tested by subjecting cultures to heat treatment at 60 °C for 30 min, followed by viability assessment on agar plates [21].

### 4.6. Haemolysis Determination

A 10 μL aliquot of bacterial suspension was spotted onto TSA supplemented with 5% defibrinated sheep blood (Thermo Fisher Scientific, Thebarton, Australia). Plates were incubated at 37 °C for 24 h. Haemolysis was evaluated based on the appearance of zones surrounding bacterial growth: complete clearance (β-haemolysis, indicating strong red blood cell lysis), partial/greenish discolouration (α-haemolysis, indicating partial lysis), or no visible change (γ-haemolysis, indicating absence of haemolytic activity) [25].

### 4.7. Antimicrobial Susceptibility Assay

Bacteria were cultured to the early log phase (OD_600_ ≈ 0.1 or equivalent to a 0.5 McFarland standard) in MHB by inoculating 1–3% of an overnight culture and incubating at 37 °C, 200 rpm. Once the cultures had reached their OD, they were further diluted 1:100 to achieve a final cell concentration of 1.5 × 10^6^ CFU/mL. Antimicrobials tested included vancomycin, gentamicin, kanamycin, streptomycin, erythromycin, tetracycline, and chloramphenicol [26], using two-fold serial dilutions from 16 μg/mL to 0 μg/mL. The diluted bacterial cultures and antimicrobials were combined in a final volume of 200 μL per well in 96-well microtiter plates. Plates were incubated statically at 37 °C for 16–20 h, and absorbance was measured at 600 nm using a SpectraMax iD5 plate reader (Bio-Strategy, Melbourne, Australia). The MIC was defined as the lowest antimicrobial concentration in wells with OD_600_ < 0.001.

### 4.8. Acid and Bile Salt Tolerance Assays

Spore suspensions were heat-treated at 60 °C for 30 min to eliminate vegetative cells and then cooled to room temperature. Subsequently, 2% of the heat-treated suspensions were inoculated into TSB, pH 4, or TSB supplemented with 0.3% bile salt. Cultures were incubated at 37 °C, 200 rpm, and samples were collected at 0, 2, and 4 h. At each time point, 100 μL was plated on TSA to assess viability. Colony confluency after 16–24 h of incubation was evaluated to determine spore survival under acidic and bile stress conditions. The method was adopted from AlGburi et al. with modifications [28].

### 4.9. Taxonomic Identification by Sanger Sequencing

Single colony of candidates 1–3 was resuspended in PrepMan Ultra buffer (Thermo Fisher Scientific, Thebarton, Australia) and submitted to Australian Genome Research Facility (AGRF) for 16S rRNA gene Sanger sequencing using the 16S microbial screen assay. Taxonomic identification was performed by AGRF, where the 16S rRNA gene was amplified and Sanger-sequenced. The resulting sequences were aligned against custom database called combined 16S database (version dated 20 Feb 2017) using Basic Local Alignment Search Tool (BLASTn, 2.17.0) to determine the closest taxonomic assignment.

### 4.10. Enzyme Activity Assays

*Bacillus* cultures (10 µL) were spotted onto skim milk agar (protease) or starch agar (amylase) (Table S4). Plates were incubated at 37 °C for 24 h. Protease activity was determined by clear zones on milk agar, indicating casein hydrolysis. Amylase activity was assessed by flooding starch agar with iodine; clear halos indicated starch degradation.

### 4.11. Agar Diffusion Assay

Pathogenic bacteria, including *E. coli*, *P. aeruginosa*, *S. aureus*, *C. perfringens*, and *S. enterica*, were cultured to the early log phase in MHB, diluted 1:20 in molten Mueller Hinton agar (MHA), and poured into petri dishes. After the agar solidified, 10 μL of the overnight *Bacillus* cultures were spotted onto the pathogenic MHA plates. The plates were incubated at 37 °C for 16–24 h. Clear zone around the spot indicated antimicrobial activities [41].

### 4.12. Preparation of Aqueous Crude Extracts

*Bacillus* candidates were cultured in TSB for 16 h at 37 °C, then they were centrifuged at 10,000× *g* for 20 min at 4 °C. Supernatant was collected and freeze-dried, then reconstituted in water and filter-sterilised to obtain the aqueous crude extracts.

### 4.13. Preparation of Ethyl Acetate (EtOAc) Extracts

*Bacillus* candidates were cultured in TSB for 16 h at 37 °C, then they were homogenised and centrifuged at 10,000× *g* for 20 min at 4 °C. The resulting supernatant was extracted with an equal volume of EtOAc three times using a separation funnel. The combined EtOAc phases were concentrated to dryness using a rotary evaporator, and the residue was dissolved in DMSO to obtain the EtOAc extracts.

### 4.14. Broth Microdilution Assay

Pathogenic bacteria, including *E. coli*, *P. aeruginosa*, *S. aureus*, *C. perfringens*, and *S. enterica*, were cultured to the early log phase in MHB, diluted 10 times, and stored on ice until further use. Aqueous crude extracts and EtOAc extracts were serially diluted in MHB in 96-well microtiter plates. An equal volume of pathogen suspension was added to each well to achieve the final working concentrations. Gentamicin (1 μg/mL) was used as the positive control, and MHB was used as the blank. Plates were incubated in a static incubator at 37 °C for 16 to 18 h. After incubation, resazurin (final concentration 0.05 mg/mL) was added to each well and incubated further for 4 h at 37 °C. Fluorescence was measured using a SpectraMax iD5 plate reader (Bio-Strategy, Melbourne, Australia) at excitation 530 nm and emission 590 nm to quantify metabolic activity [42].

### 4.15. Statistical Analysis

Broth microdilution assays included 3–4 biological replicates, each with three technical replicates. One-way ANOVA followed by Dunnett’s post-test was performed using GraphPad Prism 10. Data are reported as mean ± SEM.

## Figures and Tables

**Table 1:** MIC values of candidates 1 to 3 against EFSA-recommended antimicrobials.

Antimicrobials	Cut-Off Value *	Candidate 1	Candidate 2	Candidate 3
Vancomycin	4	0.125	0.125	0.25
Gentamicin	4	0.0078	0.0125	0.0625
Kanamycin	8	0.01563	1	0.5
Streptomycin	8	0.125	4–8	4–8
Erythromycin	4	0.0625	0.125	0.25
Tetracycline	8	0.0625	0.25	2
Chloramphenicol	8	2	4	4

* A minimum MIC value at which a strain is classified as susceptible (µg/mL).

**Table 2:** Percentage survival of benchmark *Bacillus* strains following exposure to acidic conditions (pH 4) for up to 4 h.

	0 h	1 h	2 h	4 h
Candidate 1	100%	100%	88%	85%
Candidate 2	100%	100%	97%	97%
Candidate 3	100%	100%	97%	95%
Benchmark	100%	95–100%	69–99%	67–98%

**Table 3:** Percentage survival of benchmark *Bacillus* strains following exposure to 0.3% bile salts for up to 4 h.

	0 h	1 h	2 h	4 h
Candidate 1	100%	100%	92%	92%
Candidate 2	100%	100%	71%	85%
Candidate 3	100%	100%	96%	60%
Benchmark	100%	91–100%	66–98%	44–98%

## Data Availability

The data for the research results can be obtained from Supporting Information.

## Supplementary Materials

The following supporting information can be downloaded at: https://www.mdpi.com/article/10.3390/md24040137/s1, Table S1: 16S rRNA gene Sanger sequencing results of *Bacillus* candidates 1; Table S2: 16S rRNA gene Sanger sequencing results of *Bacillus* candidates 2; Table S3: 16S rRNA gene Sanger sequencing results of *Bacillus* candidates 3; Figure S1: Protease and amylase activities of *Bacillus* candidates 1–3 on skim milk and starch agar plates; Figure S2: Agar diffusion assay showing antimicrobial activity of *Bacillus* candidates against pathogenic bacteria; Figure S3: Broth microdilution assay of aqueous crude extracts from *Bacillus* candidates 1–3 against pathogenic bacteria; Figure S4: Broth microdilution assay of EtoAc extracts from *Bacillus* candidates 1–3 against pathogenic bacteria; Table S4: List of solutions and media.

## Abbreviations

The following abbreviations are used in this manuscript:

AAFCO	Association of American Feed Control Officials
AGRF	Australian Genome Research Facility
AMR	Antimicrobial Resistance
ANOVA	Analysis of Variance
ATCC	American Type Culture Collection
*Bacillus* spp.	*Bacillus* species
BLAST	Basic Local Alignment Search Tool
CBS	Centraalbureau voor Schimmelcultures
CFU	Colony Forming Unit
*C. perfringens*	*Clostridium perfringens*
DMSO	Dimethyl Sulfoxide
*E. coli*	*Escherichia coli*
EFSA	European Food Safety Authority
EtOAc	Ethyl Acetate
GI	Gastrointestinal
MHA	Mueller–Hinton agar
MHB	Mueller–Hinton broth
MIC	Minimum Inhibitory Concentration
OD_600_	Optical Density at 600 nm
*P. aeruginosa*	*Pseudomonas aeruginosa*
QPS	Qualified Presumption of Safety
*S. aureus*	*Staphylococcus aureus*
*S. enterica*	*Salmonella enterica*
SEM	Standard Error of the Mean
TSB	Tryptic Soy Broth
TSA	Tryptic Soy Agar

## References

[1] FAO/WHO (2001). Health and Nutrition Properties of Probiotics in Food Including Powder Milk with Live Lactic Acid Bacteria.

[2] Hemarajata P., Versalovic J. (2013). Effects of probiotics on gut microbiota: Mechanisms of intestinal immunomodulation and neuromodulation. Ther. Adv. Gastroenterol..

[3] Latif A., Shehzad A., Niazi S., Zahid A., Ashraf W., Iqbal M.W., Rehman A., Riaz T., Aadil R.M., Khan I.M. (2024). Probiotics: Mechanism of action, health benefits and their application in food industries. Front. Microbiol..

[4] Bajagai Y.S., Klieve A.V., Dart P.J., Bryden W.L., FAO (2016). Probiotics in Animal Nutrition—Production, Impact and Regulation.

[5] EFSA (2026). Updated List of QPS-Recommended Microorganisms for Safety Risk Assessments Carried Out by EFSA.

[6] Hobot J.A., Tang Y.-W., Sussman M., Liu D., Poxton I., Schwartzman J. (2015). Chapter 2—Bacterial Ultrastructure. Molecular Medical Microbiology.

[7] Abriouel H., Franz C.M., Ben Omar N., Galvez A. (2011). Diversity and applications of *Bacillus* bacteriocins. FEMS Microbiol. Rev..

[8] Danilova I., Sharipova M. (2020). The Practical Potential of Bacilli and Their Enzymes for Industrial Production. Front. Microbiol..

[9] Mingmongkolchai S., Panbangred W. (2018). *Bacillus* probiotics: An alternative to antibiotics for livestock production. J. Appl. Microbiol..

[10] Ruff S.E., de Angelis I.H., Mullis M., Payet J.P., Magnabosco C., Lloyd K.G., Sheik C.S., Steen A.D., Shipunova A., Morozov A. (2024). A global comparison of surface and subsurface microbiomes reveals large-scale biodiversity gradients, and a marine-terrestrial divide. Sci. Adv..

[11] Hentschel U., Usher K.M., Taylor M.W. (2006). Marine sponges as microbial fermenters. FEMS Microbiol. Ecol..

[12] Bell J.J. (2008). The functional roles of marine sponges. Estuar. Coast. Shelf Sci..

[13] Webster N.S., Thomas T. (2016). The Sponge Hologenome. mBio.

[14] Zhang C., Kim S.K. (2010). Research and Application of Marine Microbial Enzymes: Status and Prospects. Mar. Drugs.

[15] Esposito R., Ruocco N., Viel T., Federico S., Zupo V., Costantini M. (2021). Sponges and Their Symbionts as a Source of Valuable Compounds in Cosmeceutical Field. Mar. Drugs.

[16] Mehbub M.F., Yang Q., Cheng Y., Franco C.M.M., Zhang W. (2024). Marine sponge-derived natural products: Trends and opportunities for the decade of 2011–2020. Front. Mar. Sci..

[17] Webster N.S., Hill R.T. (2001). The culturable microbial community of the Great Barrier Reef sponge *Rhopaloeides odorabile* is dominated by an α-Proteobacterium. Mar. Biol..

[18] Brinkmann C.M., Kearns P.S., Evans-Illidge E., Kurtböke D.I. (2017). Diversity and Bioactivity of Marine Bacteria Associated with the Sponges *Candidaspongia flabellata* and *Rhopaloeides odorabile* from the Great Barrier Reef in Australia. Diversity.

[19] Webster N.S. Sea Sponges and Their Microbes Hit the Wall at 33 Degrees. https://www.aims.gov.au/information-centre/news-and-stories/sea-sponges-and-their-microbes-hit-wall-33-degrees.

[20] Fekete T., Bennett J.E., Dolin R., Blaser M.J. (2015). *Bacillus* Species and Related Genera Other Than *Bacillus anthracis*. Mandell, Douglas, and Bennett’s Principles and Practice of Infectious Diseases.

[21] Wen J., Smelt J.P.P.M., Vischer N.O.E., de Vos A.L., Setlow P., Brul S. (2022). Heat Activation and Inactivation of Bacterial Spores: Is There an Overlap?. Appl. Environ. Microbiol..

[22] Sierig G., Cywes C., Wessels M.R., Ashbaugh C.D. (2003). Cytotoxic effects of streptolysin O and streptolysin S enhance the virulence of poorly encapsulated group A streptococci. Infect. Immun..

[23] Dabiré Y., Somda N.S., Somda M.K., Mogmenga I., Traoré A.K., Ezeogu L.I., Traoré A.S., Ugwuanyi J.O., Dicko M.H. (2022). Molecular identification and safety assessment of *Bacillus* strains isolated from Burkinabe traditional condiment. Ann. Microbiol..

[24] Golnari M., Bahrami N., Milanian Z., Khorasgani M.R., Asadollahi M.A., Shafiei R., Fatemi S.S.A. (2024). Isolation and characterization of novel *Bacillus* strains with superior probiotic potential: Comparative analysis and safety evaluation. Sci. Rep..

[25] Yasmin I., Saeed M., Khan W.A., Khaliq A., Chughtai M.F.J., Iqbal R., Tehseen S., Naz S., Liaqat A., Mehmood T. (2020). In Vitro Probiotic Potential and Safety Evaluation (Hemolytic, Cytotoxic Activity) of Bifidobacterium Strains Isolated from Raw Camel Milk. Microorganisms.

[26] Rychen G., Aquilina G., Azimonti G., Bampidis V., Bastos M.d.L., Bories G., Chesson A., Cocconcelli P.S., Flachowsky G., EFSA Panel on Additives and Products or Substances used in Animal Feed (FEEDAP) (2018). Guidance on the characterisation of microorganisms used as feed additives or as production organisms. EFSA J..

[27] Kosin B., Rakshit S.K. (2006). Microbial and Processing Criteria for Production of Probiotics: A Review. Food Technol. Biotechnol..

[28] AlGburi A., Volski A., Cugini C., Walsh E., Chistyakov V., Mazanko M., Bren A., Dicks L., Chikindas M. (2016). Safety Properties and Probiotic Potential of *Bacillus subtilis* KATMIRA1933 and *Bacillus amyloliquefaciens* B-1895. Adv. Microbiol..

[29] da Silva M.N., Tagliapietra B.L., Flores V.D., Richards N.S.P.D. (2021). In vitro test to evaluate survival in the gastrointestinal tract of commercial probiotics. Curr. Res. Food Sci..

[30] Khushboo, Karnwal A., Malik T. (2023). Characterization and selection of probiotic lactic acid bacteria from different dietary sources for development of functional foods. Front. Microbiol..

[31] Fujimori S. (2020). Gastric acid level of humans must decrease in the future. World J. Gastroenterol..

[32] Maillette de Buy Wenniger L., Pusl T., Beuers U., Lennarz W.J., Lane M.D. (2013). Bile Salts. Encyclopedia of Biological Chemistry.

[33] Erkkilä S., Petäjä E. (2000). Screening of commercial meat starter cultures at low pH and in the presence of bile salts for potential probiotic use. Meat Sci..

[34] Ali S., Alsayeqh A.F. (2022). Review of major meat-borne zoonotic bacterial pathogens. Front. Public Health.

[35] Abd El-Ghany W.A. (2021). *Pseudomonas aeruginosa* infection of avian origin: Zoonosis and one health implications. Vet. World.

[36] Badawy B., Moustafa S., Shata R., Sayed-Ahmed M.Z., Alqahtani S.S., Ali M.S., Alam N., Ahmad S., Kasem N., Elbaz E. (2023). Prevalence of Multidrug-Resistant *Pseudomonas aeruginosa* Isolated from Dairy Cattle, Milk, Environment, and Workers’ Hands. Microorganisms.

[37] Varijakzhan D., Loh J.Y., Yap W.S., Yusoff K., Seboussi R., Lim S.E., Lai K.S., Chong C.M. (2021). Bioactive Compounds from Marine Sponges: Fundamentals and Applications. Mar. Drugs.

[38] Amato A., Esposito R., Federico S., Pozzolini M., Giovine M., Bertolino M., Guida M., Manfra L., Libralato G., Zupo V. (2024). Marine sponges as promising candidates for integrated aquaculture combining biomass increase and bioremediation: An updated review. Front. Mar. Sci..

[39] Tran C., Cock I.E., Chen X., Feng Y. (2022). Antimicrobial *Bacillus*: Metabolites and Their Mode of Action. Antibiotics.

[40] Nicolas G.M. (2025). Secondary Metabolites from *Bacillus* spp. probiotics as potential treatments for multidrug-resistant pathogens: A comprehensive review. Curr. Res. Microb. Sci..

[41] Tran C., Horyanto D., Stanley D., Cock I.E., Chen X., Feng Y. (2023). Antimicrobial Properties of *Bacillus* Probiotics as Animal Growth Promoters. Antibiotics.

[42] Balouiri M., Sadiki M., Ibnsouda S.K. (2016). Methods for in vitro evaluating antimicrobial activity: A review. J. Pharm. Anal..

