# Supplementary material for: Development of an Integrated Screening Framework for Marine-Derived Bacillus Probiotics
Source: Mar Drugs. 2026 Apr 15;24(4):137. doi: 10.3390/md24040137 (PMC13117755; doi:10.3390/md24040137)
Supplement: Supplementary file 1 [file marinedrugs-24-00137-s001.zip › marinedrugs-4238071-supplementary.pdf]

Supplementary information for

Development of an Integrated Screening Framework for Marine-Derived *Bacillus* Probiotics

Yaoying Lu[1], **Xiaojing Chen**[2] and **Yunjiang Feng**[1, 3, *]

[1] Institute for Biomedicine and Glycomics, Griffith University, Parklands Drive, Gold Coast, QLD 4222, Australia; y.lu@griffith.edu.au

[2] Bioproton Pty Ltd., 55 Dulacca St, Brisbane, QLD 4110, Australia; wendy@bioproton.com

[3] School of Environment and Science, Griffith University, 170 Kessels Road, Brisbane, QLD 4111, Australia.

* Correspondence: y.feng@griffith.edu.au; Tel.: +61-(0)7-3735-8367

List of supporting information:

Table S1. 16S rRNA gene Sanger sequencing results of *Bacillus* candidates 1……………....3

Table S2. 16S rRNA gene Sanger sequencing results of *Bacillus* candidates 2………………3

Table S3. 16S rRNA gene Sanger sequencing results of *Bacillus* candidates 3……………...3

Figure S1. Protease and amylase activities of *Bacillus* candidates 1–3 on skim milk and starch agar plates. ……………..…..4

Figure S2: Agar diffusion assay showing antimicrobial activity of *Bacillus* candidates against pathogenic bacteria ……………..…...5

Figure S3: Broth microdilution assay of aqueous crude extracts from *Bacillus* candidates 1-3 against pathogenic bacteria……………………………………………………………6

Figure S4: Broth microdilution assay of EtoAc extracts from *Bacillus* candidates 1-3 against pathogenic bacteria. …………………………………………………………...............…7

Table S4: List of solutions and media……………………………………………….....8

Table S1. 16S rRNA gene Sanger sequencing results of candidates 1.

Rank	Sequence Entry	Hit Length	% Identity	E value
1	783903 Bacillus megaterium str. PEBM08010809 FJ685762.1 1..1510	725	100.000	0.0
2	767976 Bacillus sp. str. YSL09-1 AB576887.1 1..1507	725	100.000	0.0
3	749945 Bacillus megaterium str. rif200899 FJ527646.1 5..1516	725	100.000	0.0
4	747917 Bacillus subtilis str. rif200831 FJ527658.1 5..1517	725	100.000	0.0
5	701225 Bacillus sp. str. S21032 D84570.2 1..1512	725	100.000	0.0

Table S2. 16S rRNA gene Sanger sequencing results of candidates 2.

Rank	Sequence Entry	Hit Length	% Identity	E value
1	801854 Bacillus sp. str. WJ08 HM045832.1 1..1514	719	100.000	0.0
2	775632 Bacillus sp. str. YL223 HM486496.1 17..1438	719	100.000	0.0
3	773243 Bacillus pumilus str. 13635D EU741079.1 2..1512	719	100.000	0.0
4	764914 Bacillus altitudinis str. AP-MSU HM582688.1 1..1495	719	100.000	0.0
5	734403 Bacillus sp. str. BSFA18-2 FJ495142.1 1..1438	719	100.000	0.0

Table S3. 16S rRNA gene Sanger sequencing results of candidates 3.

Rank	Sequence Entry	Hit Length	% Identity	E value
1	800496 Bacillus subsp. subtilis str. 168 NZ_ABQK01000001.1 96390..97941	718	100.000	0.0
2	797121 Bacillus sp. str. Tianjin P2 GU936706.1 3..1509	718	100.000	0.0
3	794231 Bacillus subtilis str. 1369 GU726871.1 2..1478	718	100.000	0.0
4	793937 Bacillus subtilis str. NB-01 HM214542.1 4..1516	718	100.000	0.0
5	779942 Bacillus subtilis str. SYST2 GU568180.1 1..1459	718	100.000	0.0

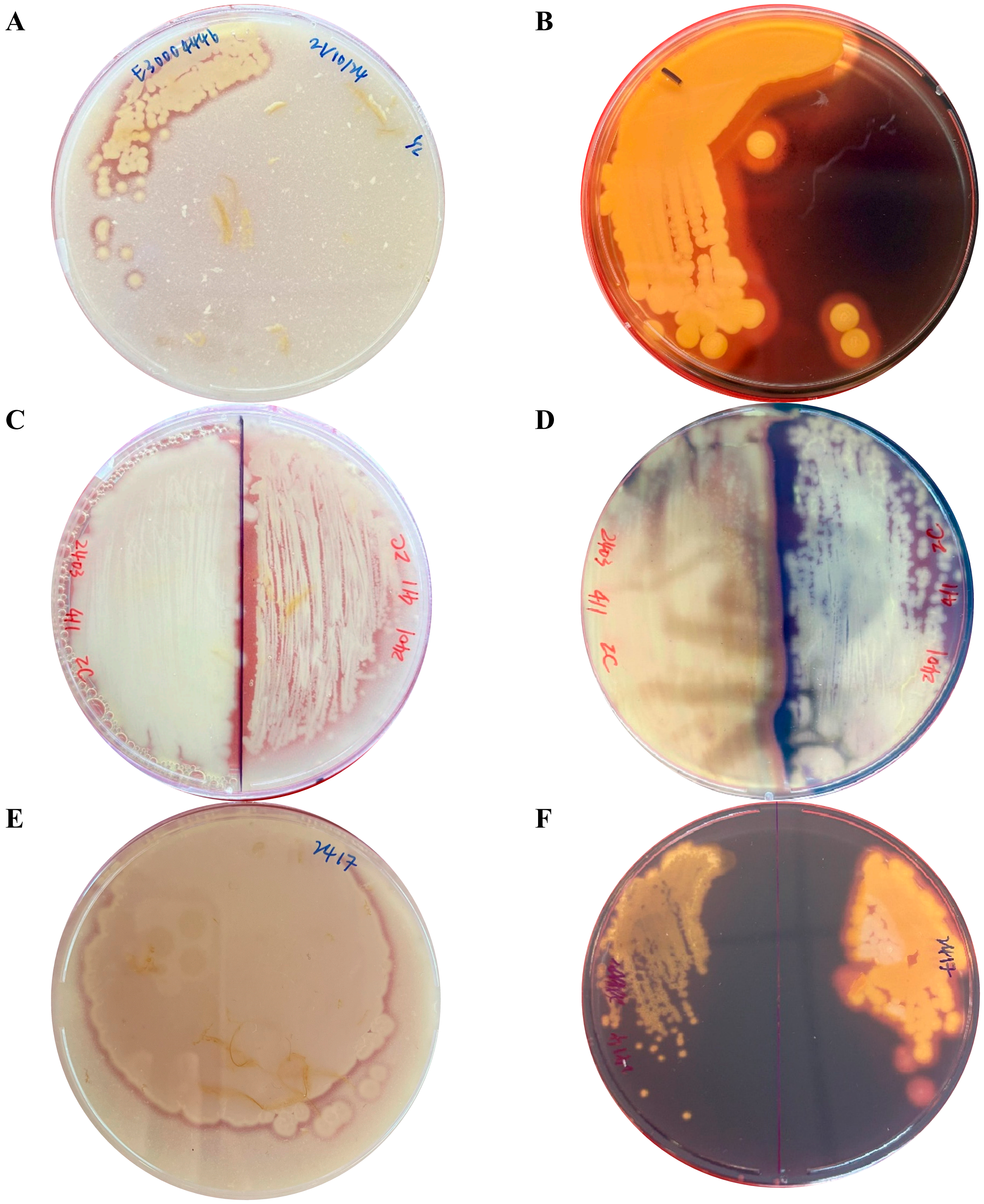

Figure S1. Protease and amylase activities of *Bacillus* candidates 1–3 on skim milk and starch agar plates. Enzymatic activity was indicated by clear zones surrounding bacterial colonies. Candidate 1 exhibited both (**A**) protease and **(B)** amylase activities. Candidate 2 showed **(C)** protease activity (right) but **(D)** no detectable amylase activity (right). Candidate 3 demonstrated both **(E)** protease and **(F)** amylase (left) activities

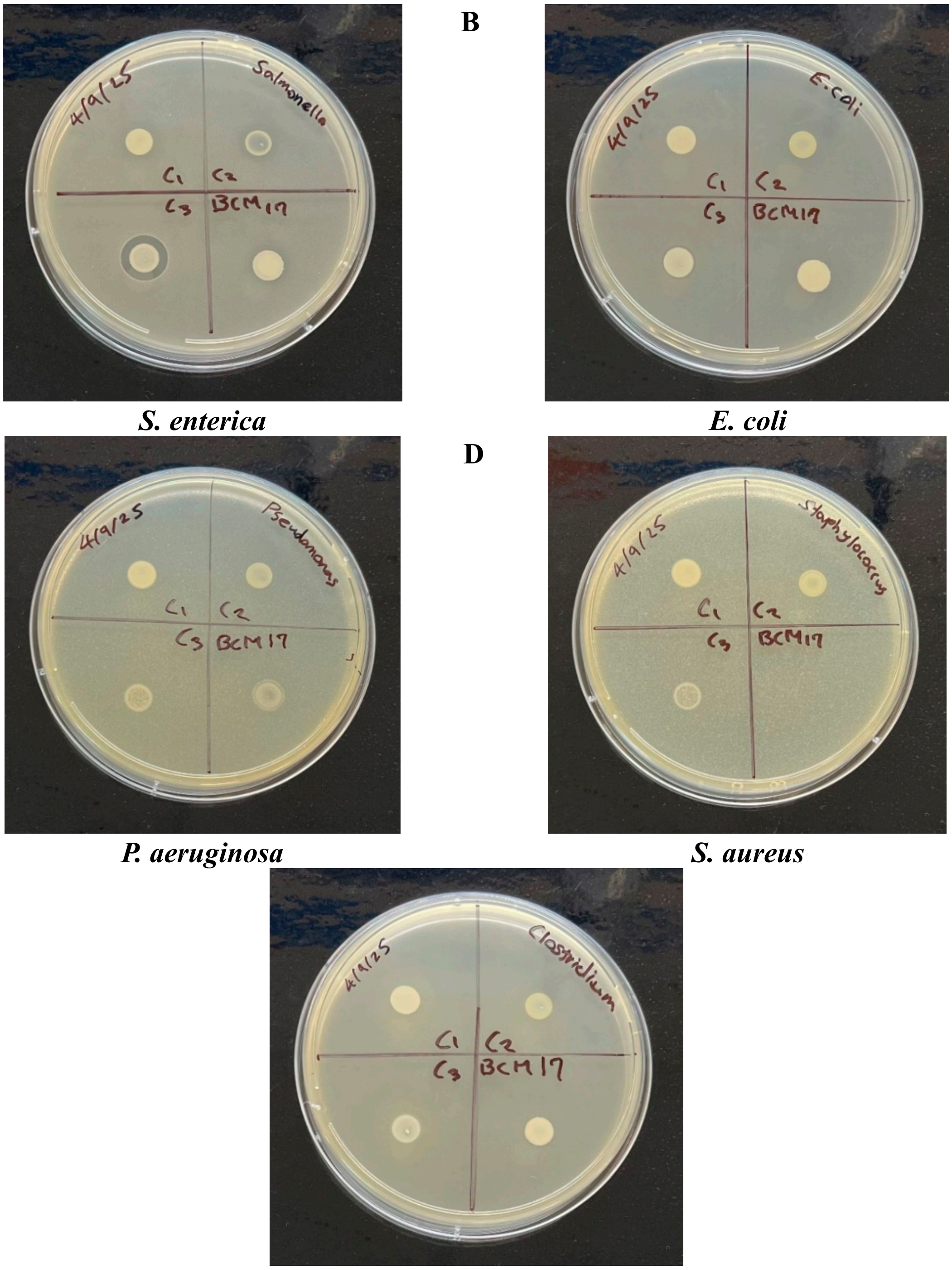

A *S. enterica*

B *E. coli*

C *P. aeruginosa*

D *S. aureus*

E *C. perfringens*

Figure S2: Agar diffusion assay showing antimicrobial activity of *Bacillus* candidates against pathogenic bacteria. **(A)** *S. enterica*, **(B)** *E. coli*, **(C)** *P. aeruginosa*, **(D)** *S. aureus*, **(E)** *C. perfringens*, were cultured in MHB and diluted 1:20 in molten MHA. Bacillus cultures, including candidates 1-3 (C1-C3) and the benchmark strain BPR17 (BCM17), were spotted onto the pathogenic MHA plates and incubated at 37 °C for 16-24 hours. Antimicrobial activity was indicated by the presence of clear inhibition zones surrounding the *Bacillus* spots.

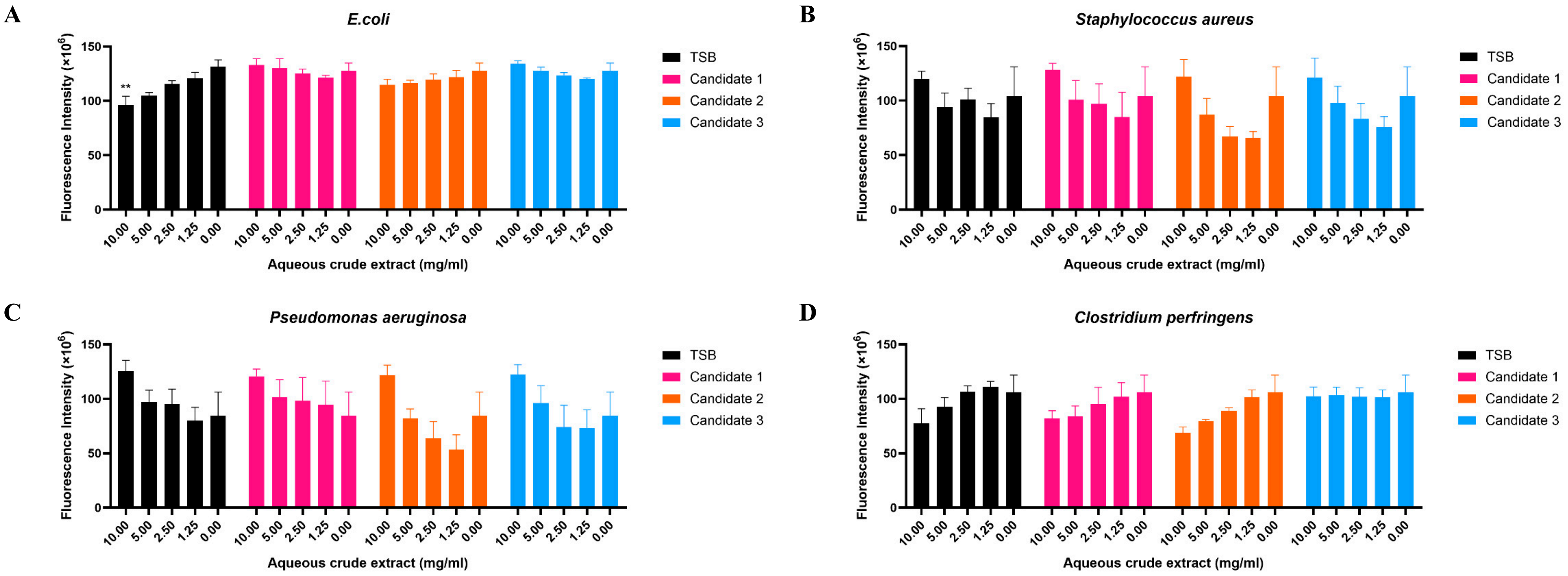

Figure S3: Broth microdilution assay of aqueous crude extracts from *Bacillus* candidates 1-3 against pathogenic bacteria. **(A)** *E. coli*, **(B)** *P. aeruginosa*, **(C)** *S. aureus*, **(D)** *C. perfringens*, were cultured in MHB and incubated with crude extracts of TSB, and candidates 1-3 for at 37°C for 16 hours. The growth of pathogenic bacteria was then assessed using Resazurin, and fluorescence intensity was measured. Mean ±SEM (n=3 or 4) of three or four independent experiments, each performed in triplicate, is shown. One-way ANOVA followed by Dunnett's test was employed to compare treated cells with untreated cells for each extract group. P**<0.01.

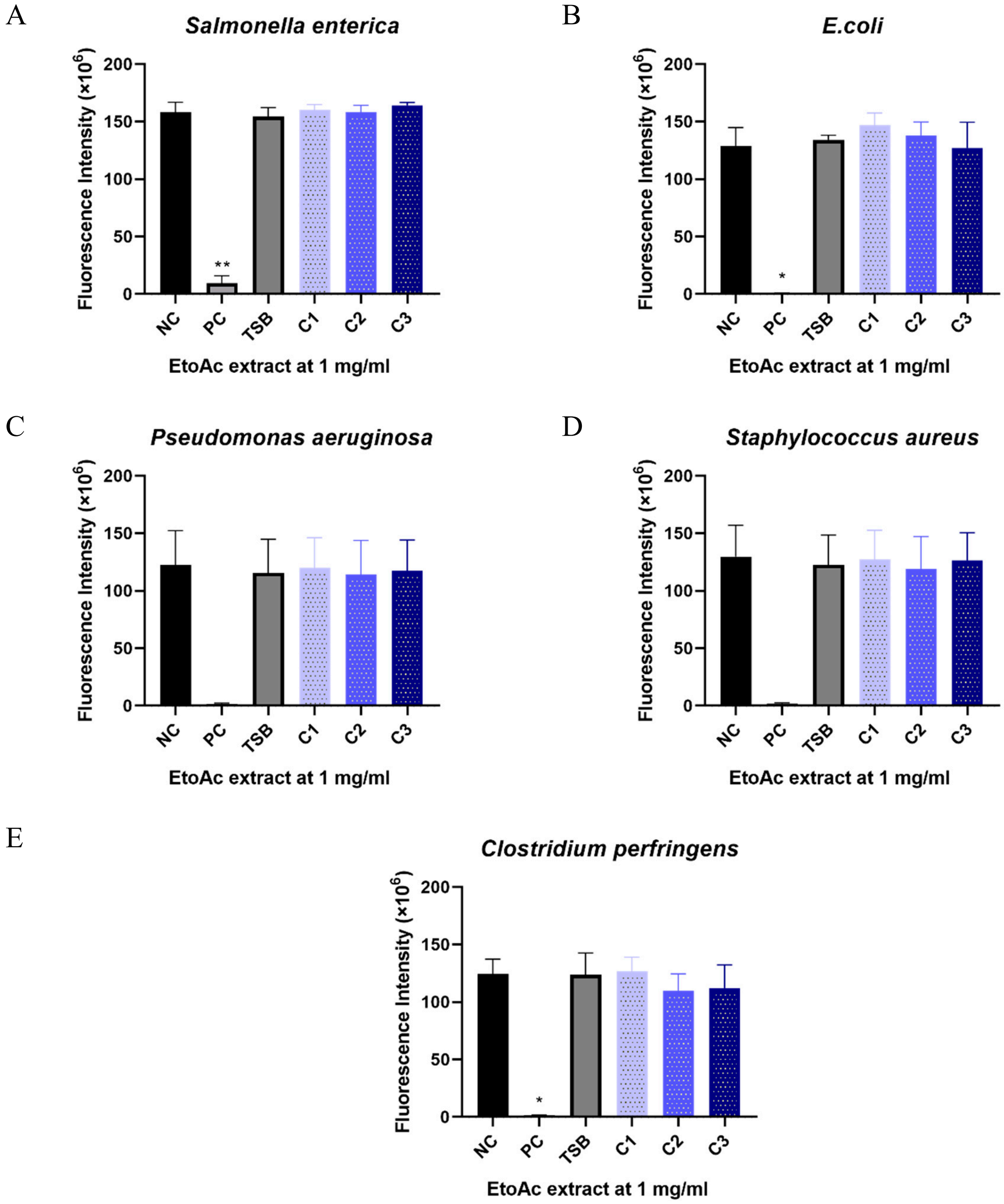

Figure S4: Broth microdilution assay of EtoAc extracts from *Bacillus* candidates 1-3 against pathogenic bacteria. **(A)** *S. enterica*, **(B)** *E. coli*, **(C)** *P. aeruginosa*, **(D)** *S. aureus*, **(E)** *C. perfringens*, were cultured in MHB and incubated with EtOAc extracts of TSB, and candidates 1-3 (C1-C3). The growth of pathogenic bacteria was then assessed using Resazurin, and fluorescence intensity was measured. NC=cells treated with 1% DMSO. PC=cells treated with 1 µg/ml gentamicin. Mean ±SEM (n=3 or 4) of three or four independent experiments, each performed in triplicate, is shown. One-way ANOVA followed by Dunnett's test was employed to compare treated cells with untreated cells within each extract group. P**<0.01.

Table S4. List of solutions and media.

Solutions/media	Composition	Use
**Tryptic soy agar (TSA)**	Tryptic Soy Broth (TSB) supplemented with 1.5% (w/v) agar.	General-purpose solid medium for routine cultivation
**Sporulation medium**	0.8% (m/v) nutrient broth, 13.4 mM KCl, 0.49 mM $MgSO_4.7H_2O$, 1 mM $Ca(NO_3)_2$, 10 µM $MnCl_2$, and 1 µM $FeSO_4$	Induction of endospores
Skim milk agar	0.3% (w/v) soluble starch, 1.0% (w/v) tryptone, 1.0% (w/v) yeast extract, 36.7 mM KH_2PO_4, and 1.5% (w/v) agar.	Detection of protease activity
Starch agar	2.8% (m/v) skim milk powder, 0.5% (m/v) tryptone, 0.25% (m/v) yeast extract, 5.55 mM dextrose, and 1.5% (m/v) agar.	Detection of amylase activity
